# X-inactive-specific transcript: a long noncoding RNA with a complex role in sex differences in human disease

**DOI:** 10.1186/s13293-024-00681-5

**Published:** 2024-12-05

**Authors:** Dan N. Predescu, Babak Mokhlesi, Sanda A. Predescu

**Affiliations:** Department of Internal Medicine, Pulmonary, Critical Care, and Sleep Medicine, Rush University Medical Center, Chicago, IL 60612 USA

**Keywords:** Sex chromosomes, XIST, gene silencing, Sexually dimorphic diseases

## Abstract

**Abstract:**

In humans, the X and Y chromosomes determine the biological sex, XX specifying for females and XY for males. The long noncoding RNA X-inactive specific transcript (lncRNA XIST) plays a crucial role in the process of X chromosome inactivation (XCI) in cells of the female, a process that ensures the balanced expression of X-linked genes between sexes. Initially, it was believed that XIST can be expressed only from the inactive X chromosome (Xi) and is considered a typically female-specific transcript. However, accumulating evidence suggests that XIST can be detected in male cells as well, and it participates in the development of cancers and other human diseases by regulating gene expression at epigenetic, chromatin remodeling, transcriptional, and translational levels. XIST is abnormally expressed in many sexually dimorphic diseases, including autoimmune and neurological diseases, pulmonary arterial hypertension (PAH), and some types of cancers. However, the underlying mechanisms are not fully understood. Escape from XCI and skewed XCI also contributes to sex-biased diseases and their severity. Interestingly, in humans, similar to experimental animal models of human disease, the males with the *XIST* gene activated display the sex-biased disease condition at a rate close to females, and significantly greater than males who had not been genetically modified. For instance, the men with supernumerary X chromosomes, such as men with Klinefelter syndrome (47, XXY), are predisposed toward autoimmunity similar to females (46, XX), and have increased risk for strongly female biased diseases, compared to 46, XY males. Interestingly, chromosome X content has been linked to a longer life span, and the presence of two chromosome X contributes to increased longevity regardless of the hormonal status. In this review, we summarize recent knowledge about XIST structure/function correlation and involvement in human disease with focus on XIST abnormal expression in males.

**Plain language summary:**

Many human diseases show differences between males and females in penetrance, presentation, progression, and survival. In humans, the X and Y sex chromosomes determine the biological sex, XX specifying for females and XY for males. This numeric imbalance, two X chromosomes in females and only one in males, known as sex chromosome dosage inequality, is corrected in the first days of embryonic development by inactivating one of the X chromosomes in females. While this “dosage compensation” should in theory solve the difference in the number of genes between sexes, the expressed doses of X genes are incompletely compensated by X chromosome inactivation in females. In this review we try to highlight how abnormal expression and function of *XIST*, a gene on the X chromosome responsible for this inactivation process, may explain the sex differences in human health and disease. A better understanding of the molecular mechanisms of XIST participation in the male-female differences in disease is highly relevant since it would allow for improving the personalization of diagnosis and sex-specific treatment of patients.

## Highlights

For years, it was believed that XIST can be expressed only from the Xi and considered a typically female-specific transcript.

Accumulating evidence indicates that XIST can also be detected in male cells.

The aberrant expression of XIST is involved in the development of human diseases by regulation of gene expression at epigenetic, chromatin remodeling, transcriptional, and translational levels.

Experimental animal models of human disease demonstrate that the males with the *Xist* gene activated display the sex-biased disease condition at a rate close to females, and significantly greater than males who had not been genetically modified. Likewise, in human, the men with supernumerary X chromosomes are predisposed toward autoimmunity like females and have increased risk for strongly female-biased diseases or compared to normal males.

Chromosome X content has been linked to a longer life span, and the presence of two chromosome X contributes to increased longevity regardless of the hormonal status.

## Introduction

The word “sex”, as defined in the Oxford English Dictionary, is “the sum of the characteristics concerned with sexual reproduction and the raising of young, by which males, females, and hermaphrodites may be distinguished”, and is used to describe the genetic and phenotypic biological, anatomical, psychological, and physiological traits that define humans as male and female organisms [1]. These traits are not exclusive but tend to differentiate male from female humans. The genetic differences between men and women beginning at conception and continuing during life, lead to sex differences in disease rate, presentation, and response to therapies. In humans, the X and Y chromosomes determine the biological sex - XX specifying for female and XY for male. However, most genes on the sex chromosomes are not implicated in sex determination, and development into a male or a female depends only on the master sex-determining locus, the *SRY* (sex-determining region Y) gene, on the male-only Y chromosome [2].

Homo sapiens is a highly sexually dimorphic species which is the result of a sexual selection on males, and a disruptive natural selection that is different in males and females. One of the best examples of sexual dimorphism in humans is the body size difference, as a reflection of sex differences in human body composition, with men having a larger lean body and muscle mass. In contrast, the females have an increase in pelvis relative to males, to accommodate increasingly large-brained neonates [3]. According to the “obstetrical dilemma” hypothesis, bipedal locomotion narrowed the human pelvis in a way that constricted the birth canal. As the size of human infants, particularly the head, is larger than their mothers’ pelvic size, selection favored wider female pelvis. This cephalopelvic disproportion and delivering large-brained infants are relevant to female obstetric adaptations [4]. This sexual dimorphism in pelvis morphology is largely the result of hormonally regulated sex-biased gene expression and becomes pronounced after puberty [5]. Thus, the sexual dimorphism comprises the natural materialization of morphological, physiological, and behavioral distinctions between males and females, added to the ones from the sex organs.

It is rationalized that the sexual dimorphism in rodents, bovines, non-human primates, and humans are the product of complex interactions amongst the sex hormones, genetic variability, and the environment. However, all these factors function on the background created by the sex determination pathway, XX or XY. The sex determination decision of XX or XY must result in sexual dimorphism throughout the whole body, from the skin to all other organs, and from the function of the brain to that of a wiggling tail. A compelling example is that of *SRY* gene, a transcriptional activator that acts exclusively on the fetal gonads to establish the synthesis of the gonadal sex hormones, which will pass the sex determination decision to every cell of the body [6]. *SRY* is considered the “master switch” for testis determination in mammals, and functions by binding to and activating the testis-specific enhancer core sequence of SRY-box 9 (*SOX9*) [7]. Once the couple of SRY-SOX9 is established, the SOX9 protein induces somatic precursor cells to develop into Sertoli cells, which orchestrate the development of the gonads as testes [8]. Without SOX9 activation, the fetal gonads develop as ovaries. It is rationalized that SRY-SOX9 interaction opposes the female-promoting regulatory network involving Wnt/β-catenin signaling that acts as a sex determinator for the XX genotype. On the other hand, the signaling on the Wnt/β-catenin pathway is first detected around E11-12 in both sexes’ bipotential gonads, acting as an anti-testicular agent by limiting the expression of SOX9 [9], but is downregulated by SRY in males. The systems of *SRY-SOX9* for male, and of *Wnt/*β*-catenin* for female determinism, are more complex as proven by the deletion of either one of two sex-specific mouse transcription factors — forkhead box L2 (FOXL2) in females, or doublesex and mab-3 related transcription factor 1 (DMRT1) in males, which could cause gonadal cells to reprogram their sex, even in adults [10, 11].

On this background, there is agreement regarding the existence of sex differences in gene expression and epigenetic profile, essentially on each human adult somatic cell, as a result of complex interactions between the sex hormones, genetic variability, and the environment [12–18]. Published data show that the expression of sexually dimorphic genes in pre-implantation development, before the new embryo produces sex hormones that are present in the circulation, is driven by sex-chromosomes based transcription, whilst later development is characterized by sex dimorphic autosomal transcription [16–18]. Thus, the emergence of sex differences prior to the development of gonads and exposure to gonadal hormones is highly suggestive of the gonadal independent contribution of sex chromosome complement [17].

In the era of genomics, the proven differences between females and males regarding longevity, disease risk, presentation, and response to therapy are accepted to have a genetic/epigenetic underpinning that operates on the background established by the sex chromosomes, XX for females and XY for males [19–21].

Even in well-controlled systems and under well-defined conditions, investigating the sexual dimorphisms of gene expression from adult tissues is complicated by the influence of gonadal hormones on the sexual background established by the sex chromosomes early on the development. This limitation was mainly overcome by the creation of the Four Core Genotypes mouse model, which allowed the scientific community to establish that the global gene expression differences stemmed from the sex chromosomes having influence over the entire genome, aka genome-wide effects [22, 23].

From early 1900, when the chromosomal basis of heredity was established by Wilson and Stevens [24, 25], more than 10 years elapsed until *Drosophila’s* eye color was confirmed as the first sex-linked trait and the molecular basis of genetics was established [26]. Subsequently, by inference, the sex-linked traits in other species were assumed to have a similar physiological basis. In humans, the traits and the diseases originating on the X, or the Y chromosome are designated as sex-linked. At the beginning of the work related to the X and Y chromosomes, when the topography of genes on the sex chromosomes and the details of their molecular regulation were established, one curious feature of X-linked genes in humans and other species was revealed - their overall expression is usually under the control of a form of gene regulation known as XIC, unique to the X chromosome.

## The good, the bad, and the ugly of XIST

**Fig. 1:**
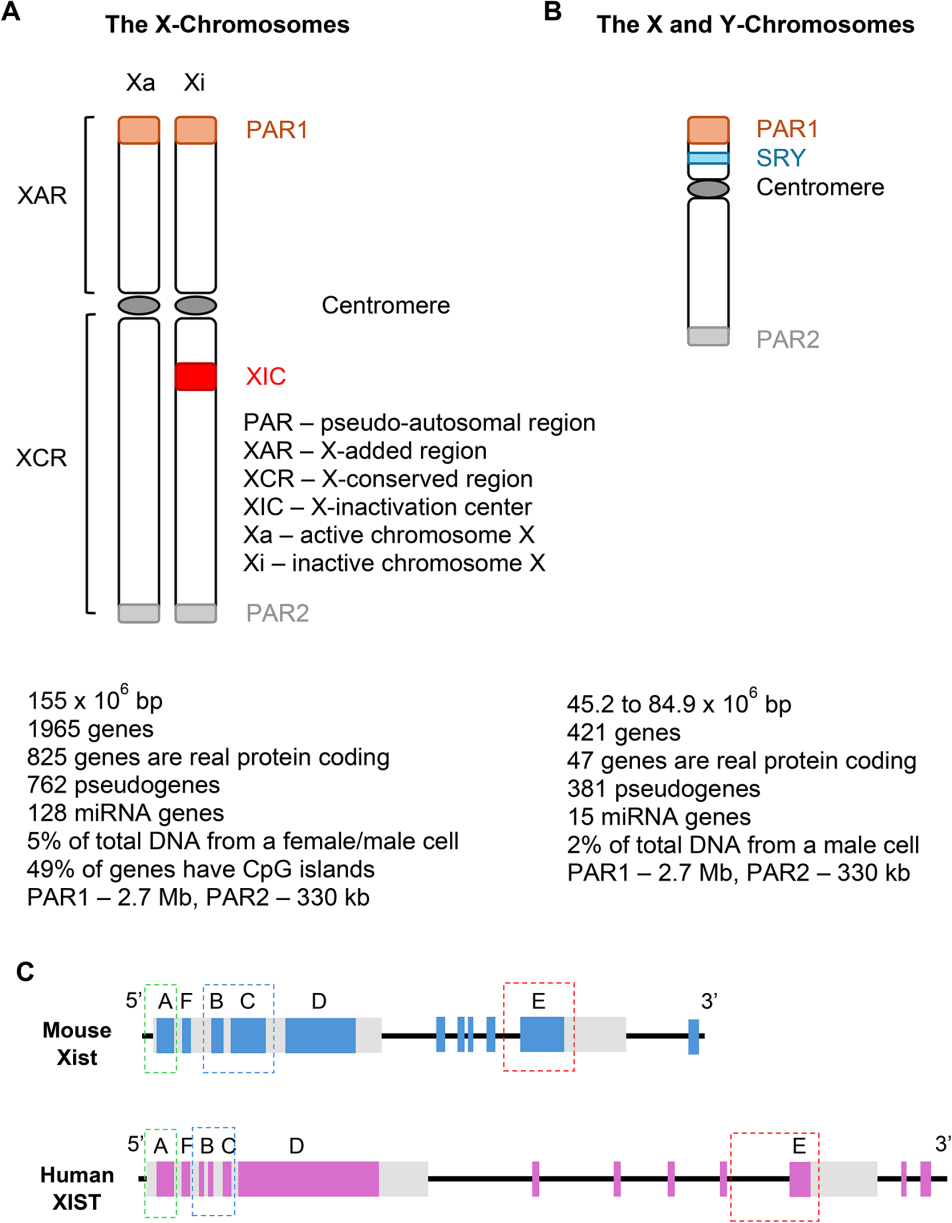
Significant features of the X and Y chromosomes. **A.** The female signature consists of two X chromosomes, the Xa and Xi pair. **B**. The Xa and one chromosome Y represent the male mark. **C.** The XIST RNA molecule repeats between human, and mouse XIST/Xist are conserved. XIST/Xist RNA contains six types of repeats, labeled A-F. The E-repeat is located at the 5 end of exon 6 of XIST/Xist and at the 5 end of exon 7 of XIST/Xist, respectively; all other repeats are contained within the large first exon of XIST/Xist. The approximate copy number and monomer length for each repeat block is: **A** repeat (mouse 8x ~ 50 nt; human 9x ~ 100 nt); **F** repeat (mouse 2x ~ 16 nt; human 2x ~ 16 nt); **B** repeat (mouse 32x ~ 7 nt; human 29x ~ 7 nt); **C** repeat (mouse 14x ~ 115 nt; human 1x ~ 115 nt); **D** repeat (mouse 10x ~ 290 nt; human 26x ~ 290 nt); **E** repeat (mouse 50x ~ 25 nt; human 28x ~ 25 nt). The dashed boxes indicate the repeats that contribute to gene silencing (green), Polycomb Group (PcG) protein recruitment (Blue) and XIST/Xist localization (red)

*The XX and XY*,* the sex chromosome complements in humans.* In eukaryotic cells, there are two X chromosomes, an X-inactive (Xi) and an X-active (Xa) in female cells, and an Xa and an Y chromosome in male cells. The Xi requires: (i) the establishment of specific post-translational modifications of histones, (ii) the creation of specific DNA methylation patterns of X-linked gene promoters, and (iii) changes in the higher-order of chromosome [27, 28]. Under evolutionary pressure, this sexual disparity in the number of X chromosomes controls both the sex-specific gene expression and the crosstalk between sex chromosomes and autosomes [29]. This is the genetic base of human sexual determinism. It is worth mentioning that even though the X chromosome accounts for 5% of active genes in humans and has a sizable contribution, it is considered as a chromosome with a low density of genes [30]. The X chromosome, with ~ 155 mega bases (mb) is 2.6-fold the size of the Y chromosome, and is “gene-rich” versus the Y chromosome, with greater than 1000 genes and 700 pseudogenes, while the Y chromosome is ~ 60 mb, is “gene-poor”, having less than 140 genes, Fig. 1, A, B [31, 32].

This numeric imbalance between the number of sex chromosomes between males and females, known as sex chromosome dosage inequality, is corrected in the first days of embryonic development by dosage compensation, a mechanisms meant to equalize sex chromosome-linked gene expression before gonadal differentiation [33]. Dosage compensation is achieved in a two-fold manner in mammals, first, by inactivation of one of the two X chromosomes in females, and second, by upregulation of X-linked genes, to balance the expression levels between X-linked and autosomal genes [34]. X chromosome inactivation is largely mediated by the lncRNA XIST, which acts to recruit repressive complexes for silencing one X chromosome. However, despite being on the Xi, about 20% of X-linked genes (termed escapees) escape from XCI and remain active. Some of these escapees have an Y chromosome paralog, and thus, they have equal expression between sexes. Other escapees are expressed exclusively from the X chromosome and will exhibit higher expression in females. Some escapees have been directly correlated to specific disease characteristics [35–37]. A less elucidated mechanism for dosage compensation to maintain a balance between X-linked and autosomal gene expression is doubling the transcription from the Xa chromosome [34].

The **Good** of XIST relates to the lyonization of gene expression. During dosage compensation, one of the X chromosomes in female cells is transcriptionally silenced at random, in the space of the X inactivation center (XIC) This phenomenon is also known as “lyonization’ in honor of Mary Frances Lyon, its first describer [38]. Lyonization starts with the upregulation of the lncRNA XIST from one allele in each cell [39–41]. XIC is about 1 Mb region of X chromosome, at Xq13.2 and contains some protein-coding genes including, *Rnf12*, as well as several lncRNAs including *RepA*,* Tsix*,* Xite*,* Jpx*,* Ftx*, and *Tsix* [28]. Whereas there is agreement that RNF12 is one of the proteins form XIC which by targeting REX1 is directly involved in XIST activation [42, 43], there are other proteins involved and the reader is referred to a recent review for an in dept analysis of XIST interaction with different proteins [44].

*Xist/XIST*, a 15 kb lncRNA in mice and 17 kb lncRNA in humans, is transcribed only from the Xi chromosome, and even if poorly conserved, XIST/*Xist* contributes and is required for XIC/Xic. Currently, because of the concept of XIST being expressed from only the Xi chromosome, it is considered a typically female exclusive transcript, and thus, not expressed in males [45]. Under normal conditions, detection of XIST presence in males was difficult, if not impossible, considering the low number of XIST molecules per cell and the analytical resolution of the in situ hybridization methodology, in the range of 100–200 kb as determined by the probe size, prevalently used for its detection [46]. The use of higher resolution qPCR and RNA sequencing significantly improved XIST detection over in situ hybridization and fluorescence [47]. XIST expression is associated with the X chromosome from which it is produced, is acting only in cis, and is a molecule created of several conserved tandem repeats labeled A to F [48]. The XIST repeats, five major structural building blocks - A, F, B/C/D, E, and exon 6/7, Fig. 1C, described for mice are conserved in their human counterpart [49, 50]. Deletion analyses of mouse Xist repeats revealed the synergistic and coordinated functions of these modules, established that XIST/Xist is a multitasking lncRNA, and helped to find out the specific RNA Binding Proteins (RBPs) that interact with the functional domains, and finally, confirmed their involvement in Xic from its beginning till at the end [51–53]. Exon 6 is considered a poor protein binding domain. A protein that is reported and confirmed to bind to the center of the Exon 6 is hnRNPU, while several proteins like: chromodomain-helicase-DNA-binding protein 4 (CHD4), enhancer of zeste polycomb repressive complex 2 subunit (EZH2), SUZ12 Polycomb Repressive Complex 2 Subunit (SUZ12), and polycomb group protein CBX (pCBX) associate with the sides of the Exon 6 [50]. Considering this distribution, a poor protein binding center domain and sides-enriched with binding proteins, it has been speculated that such scattering may help to bring its two ends to proximity, which is concordant with the spatial structure of Exon 6. Binding the hnRNPU proteins along with the BCD, a major structural domain of Xist which also binds the CDKN1A Interacting Zinc Finger Protein 1 (CIZ1), makes Exon 6 a participant in tethering Xist to Xi. Also, like the domain BCD, the compact folding of Exon 6 excludes the m^6^A methylase complex and, by this, contributes to the specificity of m^6^A modifications [50]. Thus, according to the current evidence, Exon 6 participates in three processes related to Xist structure/activity.

### Structural modularity of XIST/Xist RNA protein complexes

*Repeat A*, the focal point of XIST/Xist silencing, is highly conserved in mammals and has been shown to be indispensable for gene-silencing [54]. Repeat A essentiality for transcriptional silencing was confirmed by proteomic methodologies which mapped its RNA–protein interactions [55]. This repeat is the focal point of initiation of transcriptional silencing by recruiting SPEN (SPlit Ends), which contains the repressive domain SPOC (SPEN Paralogue/Orthologue C-terminal) that recruits the repressive factors NCoR/SMRT (Nuclear receptor Co-Repressor/Silencing Mediator of Retinoic acid and Thyroid hormone receptor) complex. After recruitment of the NCoR/SMRT complex, one of the first events that takes place is the deacetylation of histone H3 and H4. These are the first steps - recruiting and deacetylation - in initiating gene silencing. Given the biological importance of the *A* domain, repeated efforts have been made to elucidate its spatial structure to associate it with XIST biological function. Currently, the prevailing view is that repeat A functions as a nucleation center for dynamic protein assembly which organizes them as a hub of transcriptional silencing [56]. Deletion of repeat A abolishes gene silencing, which also leads to a defect in XIST/Xist local spreading and spatial exclusion of active genes from the XIST/Xist-coated nuclear compartment [57, 58]. Thus, the Repeat A domain contains RBPs involved in transcriptional silencing, splicing regulation, DNA methylation, m^6^A modification, and nuclear lamina attachment [50].

*Repeats B and C*, the heart of Polycomb Repressive Complexes mobilization, are important for locking the silent state of the Xi, through the recruitment of the PcG protein complexes to the Xi after the initial establishment of XIC by the repeat A module. Even if widely scrutinized, the mechanisms and sequences of events involved in PcG recruitment, are still an area of hot investigation [59]. As the two Repeats B and C are cytosine-reach domains, they serve as a docking site for the Heterogeneous Nuclear Ribo Nucleo Protein K (hnRNPK), which by binding the PcG Ring Finger 3/PcG Ring Finger - Protein Regulator of Cytokinesis 1 (PCGF3/PCGF5–PRC1) complex decorates the chromatin with the histone H2AK1219 mono-ubiquitination mark (H2AK119ub), which is the primary signal for recruiting the Polycomb Repressive Complex 2 (PRC2), via its recognition by Jumonji- and AT-rich interaction domain (ARID)-domain-containing protein 2 (JARID2) from the PRC2 [51]. Both repeats are binding to the hnRNPK, but it seems that the repeat C is involved in XIST localization via its interactions with the heterogenous nuclear ribonucleoprotein U/scaffold attachment factor A (hnRNPU/Saf-A) and Yin Yang 1 (YY1) [60]. While polycomb recruitment is of the essence for chromosome-wide silencing, the contribution of PRC1 and PRC2 remains to be established. So far, repeat B is a prerequisite for XIST RNA to spread and by doing this, is an active participant in gene silencing. Moreover, the deletion of repeat B or of hnRNPK showed that the recruitment of PRC1 and PRC2 is compromised. To conclude, the domains B/C are key players in heterochromatin formation and topological reconfiguration of the X chromosome and thus, in long lasting transcriptional silencing lengthwise of the Xi [59].

*Repeat D*, the neglected child of Xist/XIST, was described initially as an ~ 200 bp motif [40] and later shown to contain 10 truncated Repeat D motifs located at nucleotides ~ 5200–7900 toward the 3’ end of exon 1. It was reported to be the most complex of the Xist/XIST repeats [61]. Deletional analysis did not show any changes in phenotypes related to Xist/XIST coating or PCG recruitment in mouse embryonic fibroblasts [51]. Given the lack of functional data of the structurally complex repeat D, it has been suggested that the repeat might have a role in the early stages of XCI [59]. By comparison to the mouse, the human Repeat D is larger, suggesting that it may replace the function of repeat C, which in humans is reduced to one incomplete motif [59].

*Repeat E*, the hub of Xist/XIST localization to Xi, is important for tethering Xist/XIST by interacting with several RBPs necessary for anchoring to the Xi territory. Different methods and methodologies used to map the RBN complexes associated with each domain, established that the domain E which binds CIZ1 and hnRNPU proteins, attaches the Xist/XIST RNA to the Xi [62–64]. CIZ1 is one member of a group of matrix-associated proteins shown to interact with Xist/XIST and strongly enriched at the site of Xist/XIST RNA, in both human and mouse models [62]. Additionally, it was proposed that repeat E may participate in the recruitment of PRC2, KMT5A, and CIZ1, all of which operate independently of silencing or chromatin remodeling [65]. Furthermore, the binding of polypyrimidine Tract Binding Protein 1 (PTBP1), matrin-3 (MATR3), CUGBP (CUG triplet repeat RNA binding protein) Elav-Like Family Member 1 (CELF1), and Transactive Response DNA-binding protein 43 (TDP-43) creates a distinct functional complex which stabilizes Xist/XIST coating after the initial wave of transcriptional silencing and PcG recruitment by repeat A and repeats B/C. It is worth mentioning that, even if the polypyrimidine tract binding protein 1 (PTBP1), with TAR DNA binding protein (TARDBP), ADP-ribosylation factor 6 ( ANF622), and serine and arginine rich splicing factor 7 (SRSF7) are spatially segregated on the E domain, their functionality remains to be demonstrated [53]. By doing this, repeat E plays a central role in the maintenance of XCI likely, via the assembly of a specialized phase-separated sub-nuclear compartment necessary for the efficient maintenance of XCI [53].

*Repeat F*, the shortest of all domains, comprises two copies of 10 bp in mouse *Xist* RNA. As the deletion studies have produced conflicting results regarding its role in XCI, more work is needed to reconcile its possible roles, either as a binding site for YY 1, and/or as a participant in transcriptional silencing via the loss of tethering of Xi to the nuclear lamina [56, 66]. Recent studies reported that the Lamin B receptor binds to three sites across Xist, with the most prominent encompassing the entire F repeat, and tethers the Xi chromosome to the nuclear lamina, a region where gene expression is silenced [56]. Nuclear lamina is required for reshaping the chromatin structure to allow its proficient association with the transcriptionally active genes and silencing [67–69].

### Xist/XIST positive-negative regulators

The genes of three lncRNAs *TSIX*, *XITE* and *LINX*, whose main role is to randomly inactivate one X chromosome in the embryo development reside within the same topologically associated domain (TAD) from XIC. On the same genomic space, the Xist/*XIST* promoter shares TAD with the other three lncRNAs positive regulators, *JPX*,* FTX*,* XERT*, and the protein-Rnfl12 [70]. Outside of this brief outline of XIC, it should be mentioned that other parts within the *XIST* locus have been reported to contribute to *XIST* upregulation [71]. Work related to SPEN, essential for gene silencing during XCI, revealed a cis-acting positive feedback mechanism that boosts XIST expression by either mutual repression when *XIST* silences one or some of its cis-repressors, or by mutual activation, by *XIST* induction of cis-activators [72, 73]. The studies to understand the who and how different players involved are working together have established that the region encoding for lncRNA XIST and TSIX, the antisense transcript of Xist/XIST that can mediate its repression, are essential for initiation, establishment, and propagation of XIC [74]. There is agreement in claiming that *Tsix* is the one that dictates which chromosome X will be Xa by modifying the chromatin state of that chromosome, and by managing the DNA methylation of the *Xist/XIST* promoter [75]. However, the gene expression from corner to corner of the Xi chromosome does not happen at the pseudo-autosomal regions (PARs) that are present on both X and Y chromosomes, Fig. 1A, B [32]. On the PARs, the repressive events that mark the inactivated chromosome: (i) exclusion of RNA polymerase II, (ii) DNA methylation at promoters and (iii) adding in place histone modifications, that are missing from the PARs genes which are not imprinted [76]. As illustrated in Fig. 1A, B, in humans, PAR1 is of 2.7 Mb, while PAR2 is of only 330 kb [77]. The expression of genes from the PARs is significant because PAR1 genes are highly expressed in males, 14 out of 15 genes, making their expression to surpass the combined Xa and Xi expression in females [78].

### Xist/XIST RNA – a master regulator of XCI

*Xist/*XIST associates with 81 unique binding proteins to form different ribonucleoprotein complexes, more than 20 through direct RNA-protein interaction and others through indirect protein-protein interaction [55]. While different proteins have been described as participating in XIC, recruitment of Polycomb group proteins following Xist/XIST RNA coating occurs in the early stages of Xi establishment. Polycomb complexes PCRC1 and PCRC2 are primarily in charge of histone modifications that are paramount for XIC. While the PRC1 complex catalyzes ubiquitination of histone H2A at lysine 119 (H2AK119u1) [79], the PRC2 complex catalyzes lysine methylation -H3K27me3 [80]. Recent studies show that the non-canonical PcG RING finger 3/5 (PCG3/5)-PRC1 complex initiates recruitment of both PRC1 and PRC2 in response to Xist/XIST expression, highlighting the fact that the mechanisms of *XIST*-mediated recruitment of PcG proteins are far from being fully understood [52, 81].

Before super-resolution structural studies, it was reasoned that Xist/XIST together with its effectors proteins, other lncRNAs, and chromatin-induced modifications, accumulate over the whole chromosome and by their distribution from corner to corner, control the expression of all genes from X chromosome [82]. Super-resolution microscopy, however, has proved that XIST distributes on Xi as 50–150 nm diffraction-limited foci in differentiated cells [83]. This new concept of Xi being carried out by protein foci, despite being revolutionary, cannot explain how the known effector proteins interacting with Xist/XIST are capable of silencing more than 800 genes spanning 150 million base pairs. Nevertheless, super-resolution microscopy combined with single-particle tracking provided solid data showing that Xist/XIST foci are locally confined and once in place, they stimulate transient dynamic protein compartments formation around a slowly exchanging Xist/XIST, referred to as supramolecular complexes (SMACs). In SMACs, the rapid binding and dissociation of most Xist/XIST*-*interacting proteins is the driving force behind local protein gradients stretched over broad regions of the X chromosome responsible for silencing [84].

However, the mechanisms behind other functions of Xist/XIST remain to be revealed. The female exclusive activation of XIC is mediated by a tightly controlled balance between X-encoded XIC activators and XIC repressors [85]. Initiation of XIC is stochastic, and a negative feedback loop involving rapid silencing of some X-encoded XIC activators can prevent the inactivation of the second X chromosome [86]. The stochastic model of XIC dynamics was confirmed, at least partially, by mathematic modeling and machine learning algorithms [87]. It is assumed that once XIC is established the Xi enters the maintenance stage which is watched over by several molecular mechanisms: (i) continuous XIST expression, (ii) histones hypoacetylation, (iii) DNA methylation, and (iv) acquisition of histone macroH2A and CULLIN3/SPOP ubiquitin E3 ligase in the late stages of Xi establishment. Once established, XIC is stably propagated, but it was shown that can be reversed in vivo and in vitro by pluripotent reprogramming and by DNA hypomethylation [88, 89]. The developmentally orchestrated process of XIC is considered perpetual for the lifespan of female cells and creates a rule according to which only one Xa chromosome is typically present in human cells. However, this genetic dogma does not apply to cancerous cells of both sexes when supernumerary X chromosomes are detected and XIST expression is dysregulated. While the “re-apparition” of XIST in somatic cells is well-documented for some forms of cancers, in several autoimmune and neurological diseases, its presence in females, and particularly in male PAH patients, is a novelty [90, 91].

### The silenced state of the Xist/XIST gene

 While for females (both mice and humans) the long-noncoding RNA Xist/XIST is indispensable for initiating and maintaining XCI of one of X chromosomes during early development, the expression of XIST in male normal tissues is barely detected, but not zero. Xist/XIST presence was demonstrated by qPCR in the lung tissue of an experimental murine model of PAH, in the lung tissue of human PAH patients, and in human lung endothelial cells from lung PAH explants [92], as well as by sequencing in different human cancer tissues [93]. Significantly, XIST has also been detected in 0.6% of male somatic tissue adjacent to the tumors, presumed to be normal, and in 0.2% of normal male tissues. Hence, the values of XIST in normal somatic tissues are small, but they show that either XIST shutting off after the dosage compensation completion is not absolute, mainly for female cells, or that *XIST* gene can be reactivated in somatic cells, in both male and female, by mechanisms remaining to be elucidated. Even if the X chromosome from the male is Xa, under the circumstances described above or in various settings just starting to be identified, XIST can be detected in males’ somatic cells.

The **Good** of Xist/XIST is also found in sex-specific regulation of aging. Aging in living organisms, also known as senescence, is generally described as a cumulative, irreversible process resulting in decreased function and increased risk of death [94]. Aging is accompanied by alterations in metabolism, body composition, hormone status, and accumulation of abdominal fat. Studies of sex differences in longevity indicate a 4–10 years longer life in females versus males [95]. Using the Four Core Genotypes mouse model, Davis et al., have shown that chromosome X content has been linked to a longer life span and the presence of two chromosomes X contributes to increased longevity regardless of the hormonal status [96]. Recent studies demonstrate a female-specific feature of hypothalamic aging [97]. Using single nuclei sequencing of the aging female mouse (19–24 months) hypothalamus and bioinformatics, Hajdarovic and colleagues found that the major cell types of the hypothalamus undergo widespread transcriptional changes. The hypothalamus is a critical brain region for the regulation of physiological homeostasis, including feeding, metabolism, and sleep. The most upregulated genes included the female specific XIST and *TSIX*. Upregulation of Xist expression with age was not detected in male mice. Moreover, a learning machine approach indicated that X chromosome gene expression is sufficient to predict cellular age, and Xist is the second most important predictor of age.

A different potential explanation for the females longer life compared to males may be due to the suboptimal mitochondrial function in males and the antagonist pleiotropy of gene function between the sexes [94]. Both the mitochondrion genome and the X chromosome are asymmetrically inherited in mammals – in the sense that through evolution, the associated genes spend relatively more time under selection in females and thus, expected to be better optimized for function in females than in males and to contribute preferentially to the aging phenotype.

The **Bad** of XIST is found in the XIST driven pathologies. Many recent studies confirmed XIST participation in a substantial number of pathologies like neurological, cardiovascular, lung, kidney, pancreas, skin, autoimmune, bone diseases and cancers [91, 92, 98–101]. XIST as a nuclear regulatory RNA, not only dictates the process of dosage compensation by establishing and maintaining XCI, but also may influence gene expression by epigenetic management and as a transcriptional regulator, via RNA processing and chromatin organization. Dysregulation of its expression and defects in its functions can cause severe phenotypes and increase susceptibility to genetic disease.

### XIST and autoimmune diseases

While females have an enhanced capability to solve infections when contrasted to males, they do have an increased vulnerability to developing autoimmune diseases [102]. Autoimmune diseases, occurring in 3–5% of the population are behind the heart disease and cancer, as the third most prevalent human disease category affecting females more than males; 4 out of 5 patients are female [101]. More than 70–80% of sex-biased autoimmune diseases are female predominant, well-described examples being systemic lupus erythematosus, scleroderma, Sjogren’s syndrome, and rheumatoid arthritis. It is noteworthy to mention that in systemic lupus erythematosus, the ratio of patient sex is 9:1 female/male, and in Sjogren’s disease is 19:1 female/male [103]. Female prevalence is also observed before puberty or in postmenopausal women, when the level of sex hormones is low, implying that other mechanisms may be involved [104].

For decades, the female bias in autoimmune diseases was credited to sex hormones [105]. The females’ estrogens and males’ androgens can influence the maintenance, development, and effector functions of various immune cell subsets from the innate and adaptive immune system [105]. Recent studies strongly suggest that genetic mechanisms determined by the sex chromosomes are potentially contributors to the sex-differences in immune responses across a wide range of ages [106]. Previous work proposed that XIST may be responsible for sex bias in systemic lupus erythematosus and rheumatoid arthritis, mainly because of the dynamic regulation of XIST/Xist in lymphoid cells from humans and mice [107]. Some inconsistencies related to XIST regulation were reported, mainly during B cell development. As cells differentiate into mature follicular naïve B cells, they display a progressive loss of the XIST compartment and heterochromatin marks [108]. Even though XIST is continuously transcribed and still diffusely present in the nucleus, given the sensitivity of the RNA-fluorescence in situ hybridization methods used, the observation is not yet settled.

More recent evidence demonstrates that the pattern recognition of Toll-like receptors 7 and 8 (TLR7, TLR8), both encoded by X-linked genes, are key molecules at the intersection between the enhanced protective and autoreactive immune responses in women. Both genes escape X chromosome inactivation leading to enhanced protein expression in women compared to men [109–111]. TLR7 is mainly expressed in plasmacytoid dendritic cells, monocytes and B lymphocytes while TLR8 is preferentially expressed in monocytes, myeloid dendritic cells and neutrophils [112]. Single cell analyses of TLR7 allelic expression complemented by functional studies demonstrated that the B cells of 46, XX females and 47, XXY males are biallelic for *TLR7*, have a higher cellular expression of TLR7 protein and increased responsiveness to TLR7 ligands [109]. Recent studies by Crawford identified the lncRNA XIST as a sex-bias source of TLR7 ligands in systemic lupus erythematosus [113]. XIST RNA levels were high in the blood leukocytes of females with systemic lupus erythematosus versus controls and correlated positively with increased production of interferon-α and disease activity. Moreover, the studies demonstrated that XIST acts as an inducer of interferon-α, rather than as an interferon stimulated gene, a finding consistent with a pro-inflammatory role of XIST in systemic lupus erythematosus.

*TLR7* and *TLR8* form a co-regulated gene cluster on the X chromosome with sex specific divergent transcriptional patterns in monocytes and CD4 + T and their co-dependent transcription on the active X was observed in women and men with Klinefelter syndrome lymphocytes [106]. The elevated levels of XIST in men with Klinefelter syndrome or women with triple X syndrome indicate that XIST inactivates the supernumerary X chromosomes, but the inactivation process is incomplete, strongly suggesting a disease-augmenting role of XIST, outside of its established role in X chromosome inactivation [114].

XIST-ribonucleoprotein complex is an important driver of sex-biased autoimmunity [101]. Dou and colleagues demonstrated that the inducible transgenic expression of a non-silencing form of Xist in male mice introduced Xist ribonucleoprotein complexes and sufficed to produce autoantibodies. Xist expression in males reprogrammed T and B cell populations and chromatin states to more resemble wild-type females and promote multi-organ autoimmune pathology. Moreover, single cell gene expression analysis identified clusters of atypical B cells that accumulate as a consequence of XIST ribonucleoprotein expression.

XIST involvement in sex-biased autoimmune diseases is further supported by individuals with supernumerary X chromosomes, such as Klinefelter syndrome, a genetic condition that occurs in males when they have an extra X chromosome (47, XXY). These individuals with two X chromosomes are predisposed toward autoimmunity similar to females (46, XX), and have an increased risk of strongly female biased diseases such as Sjogren’s syndrome, systemic lupus erythematosus, and rheumatoid arthritis, compared to 46, XY males. The risk of systemic lupus erythematosus in men with Klinefelter syndrome is predicted to be similar to the risk of normal women with 46, XX, and about 14-fold higher than in men with 46, XY, consistent with the idea that systemic lupus erythematosus susceptibility can be explained, at least in part, by an X-chromosome gene-dose effect [27, 115]. Furthermore, the prevalence of systemic lupus erythematosus in females with Turner syndrome (45, X) is significantly lower compared with female trisomy (47, XXX) [27]. These observations further confirm the X chromosome gene-dose effect.

Elegant genetic system allowing for the comparison between XX *SRY* + mice genetically modified by autosomal insertion of the testes determining *SRY* transgene in a XX female sex chromosome background, and XY in a male hormonal background indicated that the two X chromosomes promoted autoimmune disease independent of hormones, strongly suggesting the lncRNA XIST may possess a risk for autoimmunity [27, 116].

Support for Xist as a major driver of autoimmune risk irrespective of sex or hormonal status in mice came from studies of NZB/W F1 mice, a spontaneous mouse model of lupus that shared similarities with human patients [117]. Remarkably, 100% of female mice develop symptoms of lupus disease around 5–6 month of age, compared to less than 40% male mice. Bone marrow transplantation experiments in which female NZB/W F1 cells were transplanted into irradiated male NZB/W F1 mice resulted in 100% male mice developing lupus symptoms, consistent with a hormone-independent role for the X chromosome in the developing of lupus disease. Even if in this context, the female predisposition has been linked to X-linked immune gene dosage, an inducible transgenic expression of a non-silencing form of Xist in male mice sufficed to produce autoantibodies [104, 106]. The prevalent consensus of Xist/XIST involvement in autoimmune diseases is that the escape from XCI in female, creates notable cellular heterogeneity and diversity in X-linked gene expression. This changed genetic background set up a matchless female’s cellular mosaicism, which provides a particular advantage compared to males, particularly during viral infections.

Taken together, this brief overlook of Xist/XIST involvement in autoimmune diseases strongly suggests that understanding the genetic mechanisms by which biological sex contributes to the strength and magnitude of innate and adaptive immunity may have implications for the treatment of these immunopathological disorders for promoting most selective protective immunity during the immune response, and for improving vaccine efficacy in both sexes.

### Xist/XIST in PAH

Pulmonary arterial hypertension (PAH), a dreadful progressive disease of lung vasculature, with a 3-year survival rate of less than 60% is the most sex-biased form of hypertension once disease penetrance, presentation, and progression are considered [118]. The sexual dimorphism of PAH should be viewed with a different lens, as all the main characteristics of the disease are 2- to 4-fold augmented in females across all races, ethnicities, and ages [119]. The widespread remodeling of the pulmonary artery vascular bed (arteriole below 120 μm diameter), and the development of hallmark plexiform lesions are the major characteristics of PAH vasculopathy, leading to right heart failure and death [120, 121].

Until recently, despite the higher susceptibility to the disease, the better female survival prognosis compared to male PAH patients was attributed to the cardio-protection mediated by female sex hormones. Recent research, however, using experimental cell models and animal-induced PAH models strongly suggests that genetic and epigenetic factors are responsible for sex differences in PAH. As different cell types isolated from the lung explants of PAH patients maintain their in vivo characteristics (i.e., apoptosis-resistance, hyperproliferation, inflammatory phenotype, etc.,) or preserve cellular identity by using their cell progenitors, they were valuable tools in obtaining evidence for the sexual dimorphism of PAH [122, 123]. The X chromosome is home to several important genes associated with PAH pathobiology [119]. Recent studies have demonstrated increased expression and activity of XIST/Xist induced by an intersectin-1 protein fragment with proliferative potential (EH_ITSN_), in female PAH lung specimens, as well as a murine model of PAH that closely recapitulates the human disease [90, 92]. This increased expression of XIST/Xist leads to increased expression and activity of the X-linked gene *Elk1* and endothelial cell hyper-proliferation [92]. When male PAH specimens were analyzed, a wide range of XIST expression levels were detected [91]. Pulmonary artery endothelial cells of PAH male patients showed a wide range of XIST expression, with the highest XIST level over 16-fold greater than the lowest XIST level; on average 10.3-fold increase in high XIST versus low. DNA methylation status in the XIST promoter region and the lncRNA TSIX expression/methylation was proposed to explain, at least in part the significant variability of XIST expression in male EC_PAH_. Also, the studies found an important association between XIST levels and endothelial cell proliferation via a crosstalk between the EH_ITSN_-triggered p38/Elk1/c-Fos pathogenic proliferative signaling and XIST-EZH2-Klf2 interaction [91]. Unlike female endothelial cells, where higher XIST levels correlated to more proliferative endothelial phenotype, in males PAH patients, endothelial cells with low XIST were more proliferative than the endothelial cells with high XIST, suggesting that in male patients, augmented XIST expression may provide protection against endothelial hyperproliferation, consistent with a sex-specific relationship between XIST abnormal expression and diseases.

The knowledge of the functional impact of somatic XIST perturbation in human male cells is limited [93]. Moreover, the correlation between *XIST* and *TSIX*, an antisense *XIST* gene, is largely unexplored. It has been suggested that the lncRNA *TSIX* transcription regulates chromatin conformation of the *XIST* locus, causing a repressive chromatin structure [75, 124]. While in mice, the lncRNA *Tsix* is transcribed over the *Xist* locus in the antisense orientation and functions as a repressor of *Xist* on the chromosome from which it is transcribed, this mechanism is not yet demonstrated in humans and is still controversial [125, 126]. Interestingly, pulmonary artery endothelial cells of PAH male patients with high XIST levels belong to hypoxic patients, suggesting a possible link between XIST levels and hypoxic signaling in PAH.

Recent developments revealed significant crosstalk of XIST, via a range of miRNAs, with proteins that affect the pathological mechanisms of cancer and cardiovascular diseases including PAH. XIST modulates via the miR 93-5p the HIF1A-Axl axis in colorectal cancer [127]. Using both male and female colorectal cancer samples, Yang demonstrated that XIST is the competitive endogenous RNA of miR93-5p to promote HIF-1 A and then the upregulated AXL level facilitates migration and proliferation of colorectal cancer. HIF1A is a master regulator of oxygen homeostasis with augmented expression in PAH [128–131], while AXL is a key regulator of endothelial BMPR2 signaling, whose loss-of-function mutations is the most common genetic cause of PAH [132, 133].

In vitro studies using myocardial H9c2 cells demonstrated that XIST provides protection against injury induced by hypoxia and regulates myocardial infarction via the mir486-5p/Sirtuin 1 axis [134]. Sirtuin 1 is a nicotinamide adenine dinucleotide (NAD+)-dependent histone deacetylase and a regulator of endothelial cells and smooth muscle cells proliferation in PAH [135, 136].

Kostyunina et al., reported sexual dimorphism in the development and progression of PAH associated with significant differences in RNA and protein expression between male and female pulmonary microvascular endothelial cells grown in conditions of physiological shear stress in response to hypoxia; these differences were independent of the sex hormone environment [137].

In vitro studies, using mouse lung endothelial cells of male and female wt- and parkin-/- mice have shown epigenetic sex differences (methylation, histone modification), and effects on gene expression in experimental pulmonary hypertension that may account for the sexual dimorphism found in vivo [138]. Xist/XIST in mammals regulates mitochondrial maintenance across generations and in aging [94]. Asymmetric inheritance of mitochondrial genes and sex chromosomes promotes the evolution of sexually antagonistic gene functions and are expected to contribute preferentially to the aging phenotype [94]. Thus, in evolution, the mitochondria may be less optimized for function in the male vs. the female, and this sub-optimal function of mitochondria in males may contribute to sex differences in stress response and apoptosis [139]. It may be attractive to speculate that while men are less susceptible to PAH, they have worse outcomes once diagnosed with the disease. Thus, Xist/XIST may be a regulator of sexual differentiation, and a co-regulator of apoptosis and life span. Its level of expression, interactions and pleiotropic effects may facilitate understanding sex differences in disease.

### XIST and obstructive sleep apnea

Obstructive sleep apnea (OSA) characterized by recurrent episodes of partial or complete upper airway collapse during sleep, is a risk factor for PAH [119, 140]. Limited studies related to XIST involvement in OSA demonstrated that XIST expression was significantly increased in the adenoids of patients with OSA compared to healthy controls [141]. The OSA group included 19 females and 7 males, while the healthy group included 19 females and 2 males. XIST expression negatively correlated with the expression of glucocorticoid receptor α; XIST increased the production of inflammatory cytokines including IL-8, TNFα, IL-6 and IL-1β, in an NF-kB-dependent manner. These findings suggest that XIST /glucocorticoid receptor α/NF-kB signaling contributes to the inflammation in the adenoids of patients with OSA.

Men with supernumerary X-chromosomes, such as individuals with Klinefelter syndrome (47, XXY) have a higher risk of developing OSA [142–146]. Genetic variants of the Klinefelter syndrome (48, XXYY) also showed a history of OSA and obesity [147]. While the additional chromosome X is associated with an expected significant increase in XIST, it cannot convincingly link OSA to XIST. Genome-wide methylation data showed that despite increased XIST expression in supernumerary X-chromosome syndrome, the inactivation process of the extra X chromosome is incomplete [114], leading to a global genomic imbalance affecting the male cells epigenome and transcriptome.

### XIST and obesity

Sex differences in obesity and the gonadal hormones as critical determinants of these differences are well-established concepts. However, the emergence of sex differences prior to the development of gonads and exposure to gonadal hormones is highly suggestive of gonadal independent contribution of sex chromosome complement [17, 148]. Studies using the Four Core Genotypes mouse model that allows the distinction of gonadal from sex chromosomal effects have demonstrated that X chromosome dosage influences food intake, fat accumulation and obesity related conditions [149, 150]. Several studies have revealed that visceral obesity is one of the phenotypic characteristics of men with Klinefelter syndrome [151–156]. High fat diet induced obesity aggravated the severity of lupus in TLR8 deficient female mice which develop spontaneous lupus-like disease due to increased TLR7 signaling by dendritic cells [157]. XIST/Xist expression is significantly higher in human adipose tissue of females than males, as well as in female mice homozygous for the obese spontaneous mutation (ob/ob) [158, 159]. However, currently the role of Xist/XIST in adipose tissue metabolism is still unknown.

Interestingly, XIST expression is significantly upregulated during brown adipocytes differentiation in vitro [158]. Wu and colleagues found that overexpression of XIST during differentiation increased the expression of brown adipose tissue markers genes such as *UCP1* (uncoupling protein 1), CCAAT enhancer binding protein (C/ebp) α, and PPARG (peroxisome proliferator activated receptor gamma). Overexpression of Xist in male mice, activates brown adipose tissue activity, preventing high fat diet induced obesity and improving adipose tissue function.

Recent evidence indicates that XIST is transcriptionally regulated by a glucocorticoid/ glucocorticoid receptor signaling, both in vitro and in vivo [160]. This is relevant as the glucocorticoids regulate adipogenesis and adipose metabolic function [161].

The **Ugly** of XIST concerns its involvement in carcinogenesis. A mounting body of evidence demonstrates XIST involvement in different human cancers as both a tumor suppressor gene, and an oncogene [100, 162]. Onco-suppressive properties of XIST have been reported in several hematologic malignancies. For instance, deletion of *XIST* in hematopoietic cells triggered myeloproliferative and myelodysplastic syndrome [163]. Reactivation of XIST in males with Hodkin’s disease was associated with a better prognosis [162]. XIST was found to interact with miR-92b in hepatocellular carcinoma and inhibited cancer progression mediated by its direct target, Smad7 [164].

Many studies have reported the association between XIST expression with tumorigenesis and tumor progression [93, 165]. Emerging evidence indicates a significant role of lncRNA XIST/EZH2/Klf2 axis in promoting cell proliferation and cancer aggressiveness [166]. Upregulation of XIST was reported to be associated with overexpression of EZH2, a key component of PRC2, and to act as an adverse prognosis indicator of cancer in male and female patients [167, 168]. Knockdown of XIST exerts tumor-suppressive functions in human glioblastoma stem cells [169]. Likewise, tetracycline inducible Xist transgene expression in thymic lymphoma was shown to cause tumor block by X chromosome silencing, in mice [170]. XIST dispersion was associated with cancer cells growth, particularly in breast cancer [171].

The male predominance in cancer risk and mortality has been well recognized for decades and besides smoking, alcohol, and certain occupational exposures, the sex gap and XIST involvement remains for the most part unexplained. Available data show that male bias to many cancers is frequently associated with six genes that escape or variably escape from XCI ATP-dependent helicase X-linked helicase II (*ATRX*), connector enhancer of kinase suppressor of Ras-2 (*CNKSR2*), dead-box helicase 3 X-linked (*DDX3X*), histone demethylase 5 C (*KDM5C*), histone demethylase 6 A (*KDM6A*), and melanoma antigen gene member C3 (*MAGEC3*). Loss-of-function mutations of these genes is more frequently in males than females, and thus the “EXiTS” hypothesis in which genes that *e*scape from *X i*nactivation are *t*umor *s*uppressors, was proposed. Accordingly, their expression from the Xi, is protecting females from cancer [172]. There are other genes [i.e., *DDX3X*, cyclin Q *(CCNQ*) and zinc finger protein X-linked *(ZFX*)] that escape XCI, or that are more frequently mutated in males, with *DDX3X* mutations found only in males. The same study found that from X-linked genes, 45 are upregulated in females, with *DDX3X* expression being 1.3-fold higher in female melanomas, and all of them had lower levels of promoter methylation. *DDX3X*-associated gene expression analysis pointed out that its loss is associated with de-differentiation, invasiveness, and cut down proliferation [173].

Everything sets off with the fact that XIST is not expressed in male somatic tissues, nevertheless its elevated expression was noted in males’ tumors like bladder, colorectal, and lung cancers, and correlated with shorter survival and worse prognosis [166, 174]. Thus, even in males’ tumors XIST presence is associated with tumorigenesis, metastasis, and tumor developmental stage.

The current data also show that XIST serves primarily as a miRNA molecular sponge to regulate the expression of miRNA targets in male biased cancers [175]. Outside of sponging, XIST’s direct interactions with different proteins change their functions. Among them, XIST interaction with the DNA demethylase ten-eleven translocation methyl cytosine dioxygenase 1 (TET1) reduces TET1-mediated demethylation of p53, causing an inhibitory effect on p53 expression in bladder cancer [176]. XIST also binds with the H3K27me3-specific methyltransferase EZH2, a subunit of the polycomb repressive complex 2, and by doing so, it silences the expression of Klf2, a tumor suppressor in non-small cell lung cancer.

The idea that XIST is expressed in male somatic cells under distinct conditions should be considered and exploited, and further research is needed to better understand its contribution to human disease.

## Conclusions

LncRNA XIST is a key player in many sex-biased diseases. Dysregulation of its expression and defects in its functions can cause severe phenotypes and increased susceptibility to genetic disease. Given the longstanding exclusion of the X chromosome from the genome-wide association studies [177], the roles of X-chromosomal genes, *XIST* included, in complex traits in females, are still inadequately appreciated, while for males, the implication of XIST functions and its participation and contribution to diseases states, other than cancer, remains in its infancy. Future research should focus more on the relationship between XIST expression and diseases with consideration of sex differences.

Studies to gain a deeper understanding of XIST function, and how its aberrant expression may regulate key cellular processes in a sex-dependent manner may lead to establishing XIST as a biomarker to track or predict disease progression and as a potential therapeutic target.

## Data Availability

No datasets were generated or analysed during the current study.

## Abbreviations

ATRX: ATP-dependent helicase ATRX
X: linked helicase II
BMPR2: Bone morphogenetic protein receptor 2
CUGBP: CUG triplet repeat RNA binding protein
CCNQ: Cyclin Q
CIZ1: CDKN1A interacting zinc finger protein 1
CNKSR2: Connector enhancer of kinase suppressor of ras-2
DDX3X: Dead-box helicase 3 X-linked
DMRT1: Doublesex and mab-3 related transcription factor 1
EZH2: Enhancer of zeste polycomb repressive complex 2 subunit
EH: Epsin 15 homology domain
EH_ITSN_: Intersectin-1 protein fragment with proliferative potential
FOXL2: Forkhead box L2
H2AK119ub: Histone H2AK1219 mono-ubiquitination
hnRNPK: Heterogenous nuclear ribonucleoprotein K
hnRNPU/Saf-A: Heterogenous nuclear ribonucleoprotein U/ scaffold attachment factor A
JARID2: Jumonjiand AT-rich interaction domain (ARID)-domain-containing protein 2
KDM5C: Histone demethylase 5 C
lncRNA: Xist - long noncoding RNA X-inactive specific transcript
LRGs: Genes - lncRNA genes
MAGEC3: Melanoma Antigen Gene member C3
MATR3: Matrin-3
NCoR/SMRT: Nuclear receptor co-repressor/ silencing mediator of retinoic acid and thyroid
NFkB: Nuclear factor kappa B subunit 1
OSA: Obstructive sleep apnea
PAH: Pulmonary arterial hypertension
PARs: Pseudo-autosomal regions
PcG: Polycomb group
PCGF3/PCGF5–PRC1: Polycomb group ring finger 3 / polycomb group ring finger - protein regulator of cytokinesis 1
PRC2: Polycomb repressive complex 2
PTBP1: Polypyrimidine Tract Binding Protein 1
PPARG: Peroxisome proliferator activated receptor gamma
pCBX: Polycomb group protein CBX
RBPs: RNA-Binding-Proteins
SPEN: SPlit Ends hormone receptor)
SMACs: Supramolecular complexes
SUZ12: SUZ12 Polycomb Repressive Complex 2 Subunit
TDP-43: Transactive response DNA-binding protein 43
TAD: Topologically associated domain
TLR: Toll-like receptors
TNF: Tumor necrosis factor
TET1: Ten-eleven translocation methylcytosine dioxygenase 1
UCP-1: Uncoupling protein 1
XCI: X chromosome inactivation
Xa: Active X chromosome
Xi: Inactive X chromosome
YY1: Yin Yang 1
ZFX: Zinc finger protein X-linked

## References

[1] Oxford English Dictionary Online, ed. M. Proffitt. Oxford University Press; 2024.

[2] Bachtrog D, et al. Sex determination: why so many ways of doing it? PLoS Biol. 2014;12(7):e1001899. 24983465 10.1371/journal.pbio.1001899 PMC4077654

[3] Lassek WD, Gaulin SJC. Substantial but Misunderstood Human sexual dimorphism results mainly from sexual selection on males and natural selection on females. Front Psychol. 2022;13:859931. 35664212 10.3389/fpsyg.2022.859931 PMC9156798

[4] Washburn SL. Tools and human evolution. Sci Am. 1960;203:63–75. 13843002

[5] Huseynov A, et al. Developmental evidence for obstetric adaptation of the human female pelvis. Proc Natl Acad Sci U S A. 2016;113(19):5227–32. 27114515 10.1073/pnas.1517085113 PMC4868434

[6] Schlessinger D, et al. Determination and stability of gonadal sex. J Androl. 2010;31(1):16–25. 19875493 10.2164/jandrol.109.008201 PMC2882171

[7] Kashimada K, Koopman P. Sry: the master switch in mammalian sex determination. Development. 2010;137(23):3921–30. 21062860 10.1242/dev.048983

[8] Wilhelm D, Palmer S, Koopman P. Sex determination and gonadal development in mammals. Physiol Rev. 2007;87(1):1–28. 17237341 10.1152/physrev.00009.2006

[9] Chassot AA, et al. WNT4 and RSPO1 together are required for cell proliferation in the early mouse gonad. Development. 2012;139(23):4461–72. 23095882 10.1242/dev.078972

[10] Uhlenhaut NH, et al. Somatic sex reprogramming of adult ovaries to testes by FOXL2 ablation. Cell. 2009;139(6):1130–42. 20005806 10.1016/j.cell.2009.11.021

[11] Matson CK, et al. DMRT1 prevents female reprogramming in the postnatal mammalian testis. Nature. 2011;476(7358):101–4. 21775990 10.1038/nature10239 PMC3150961

[12] Blekhman R, et al. Sex-specific and lineage-specific alternative splicing in primates. Genome Res. 2010;20(2):180–9. 20009012 10.1101/gr.099226.109 PMC2813474

[13] Dewing P, et al. Sexually dimorphic gene expression in mouse brain precedes gonadal differentiation. Brain Res Mol Brain Res. 2003;118(1–2):82–90. 14559357 10.1016/s0169-328x(03)00339-5

[14] Marks H, et al. The transcriptional and epigenomic foundations of ground state pluripotency. Cell. 2012;149(3):590–604. 22541430 10.1016/j.cell.2012.03.026 PMC3398752

[15] Werner RJ, et al. Sex chromosomes drive gene expression and regulatory dimorphisms in mouse embryonic stem cells. Biol Sex Differ. 2017;8(1):28. 28818098 10.1186/s13293-017-0150-x PMC5561606

[16] Arnold AP, Chen X, Itoh Y. What a difference an X or Y makes: sex chromosomes, gene dose, and epigenetics in sexual differentiation. Handb Exp Pharmacol, 2012;(214): pp. 67–88. 10.1007/978-3-642-30726-3_4 PMC4150872 23027446

[17] Lowe R, et al. Sexually dimorphic gene expression emerges with embryonic genome activation and is dynamic throughout development. BMC Genomics. 2015;16(1):295. 25888192 10.1186/s12864-015-1506-4 PMC4410000

[18] Nugent BM, et al. Placental H3K27me3 establishes female resilience to prenatal insults. Nat Commun. 2018;9(1):2555. 29967448 10.1038/s41467-018-04992-1 PMC6028627

[19] Engel N. Sex differences in early Embryogenesis: inter-chromosomal regulation sets the stage for sex-biased gene networks: the dialogue between the sex chromosomes and autosomes imposes sexual identity soon after fertilization. BioEssays. 2018;40(9):e1800073. 29943439 10.1002/bies.201800073

[20] Khramtsova EA, Davis LK, Stranger BE. The role of sex in the genomics of human complex traits. Nat Rev Genet. 2019;20(3):173–90. 30581192 10.1038/s41576-018-0083-1

[21] Raznahan A, et al. Sex-chromosome dosage effects on gene expression in humans. Proc Natl Acad Sci U S A. 2018;115(28):7398–403. 29946024 10.1073/pnas.1802889115 PMC6048519

[22] Arnold AP. Mouse models for evaluating sex chromosome effects that cause sex differences in non-gonadal tissues. J Neuroendocrinol. 2009;21(4):377–86. 19207816 10.1111/j.1365-2826.2009.01831.x PMC2669494

[23] Wijchers PJ, et al. Sexual dimorphism in mammalian autosomal gene regulation is determined not only by sry but by sex chromosome complement as well. Dev Cell. 2010;19(3):477–84. 20833369 10.1016/j.devcel.2010.08.005

[24] Stevens N. Studies in spermatogenesis. Part II A comparative study of the heterochromosomes in certain species of Coleoptera, Hemipteraand Lepidoptera with Special reference to sex determination. Washington: Carnegie Institute of Washington; 1906.

[25] Wilson E. The chromosomes in relation to the determination of sex in insects. Sci N S. 1905;22:500–2. 10.1126/science.22.564.500 17748139

[26] Morgan T. The mechanism of Mendelian heredity, ed. H.H.a. Company. 1915, New York: Electronic reproduction. New York, N.Y.: Columbia University Libraries. 2007.

[27] Syrett CM, Anguera MC. When the balance is broken: X-linked gene dosage from two X chromosomes and female-biased autoimmunity. J Leukoc Biol. 2019;106(4):919–32. 31125996 10.1002/JLB.6RI0319-094R PMC7206452

[28] Loda A, Heard E. Xist RNA in action: past, present, and future. PLoS Genet. 2019;15(9):e1008333. 31537017 10.1371/journal.pgen.1008333 PMC6752956

[29] Deegan DF, Engel N. Sexual dimorphism in the age of Genomics: how, when, where. Front Cell Dev Biol. 2019;7:186. 31552249 10.3389/fcell.2019.00186 PMC6743004

[30] Ross MT, et al. The DNA sequence of the human X chromosome. Nature. 2005;434(7031):325–37. 15772651 10.1038/nature03440 PMC2665286

[31] Reece J et al. The chromosomal basis of sex. Chambell Biology, 10th Edition. 2011, San Francisco, CA: Pearson.

[32] Li X. Sex chromosomes and sex chromosome abnormalities. Clin Lab Med, 2011;31(4): p. 463 – 79, vii. 10.1016/j.cll.2011.08.013 22118732

[33] Furlan G, Galupa R. Mechanisms of choice in X-Chromosome inactivation. Cells, 2022;11(3). 10.3390/cells11030535 PMC8833938 35159344

[34] Abramowitz LK, Olivier-Van S, Stichelen, Hanover JA. Chromosome imbalance as a driver of sex disparity in disease. J Genomics. 2014;2:77–88. 25031659 10.7150/jgen.8123 PMC4091450

[35] Carrel L, Brown CJ. When the Lyon(ized chromosome) roars: ongoing expression from an inactive X chromosome. Philos Trans R Soc Lond B Biol Sci, 2017;372(1733). 10.1098/rstb.2016.0355 PMC5627157 28947654

[36] Posynick BJ, Brown CJ. Escape from X-Chromosome inactivation: an evolutionary perspective. Front Cell Dev Biol. 2019;7:241. 31696116 10.3389/fcell.2019.00241 PMC6817483

[37] Qi S, et al. X chromosome escapee genes are involved in ischemic sexual dimorphism through epigenetic modification of inflammatory signals. J Neuroinflammation. 2021;18(1):70. 33712031 10.1186/s12974-021-02120-3 PMC7953638

[38] Lyon MF. Gene action in the X-chromosome of the mouse (Mus musculus L). Nature. 1961;190:372–3. 13764598 10.1038/190372a0

[39] Brown CJ, et al. Localization of the X inactivation centre on the human X chromosome in Xq13. Nature. 1991;349(6304):82–4. 1985270 10.1038/349082a0

[40] Brown CJ, et al. The human XIST gene: analysis of a 17 kb inactive X-specific RNA that contains conserved repeats and is highly localized within the nucleus. Cell. 1992;71(3):527–42. 1423611 10.1016/0092-8674(92)90520-m

[41] Gendrel AV, Heard E. Noncoding RNAs and epigenetic mechanisms during X-chromosome inactivation. Annu Rev Cell Dev Biol. 2014;30:561–80. 25000994 10.1146/annurev-cellbio-101512-122415

[42] Barakat TS, et al. RNF12 activates Xist and is essential for X chromosome inactivation. PLoS Genet. 2011;7(1):e1002001. 21298085 10.1371/journal.pgen.1002001 PMC3029249

[43] Wang F, Mehta P, Bach I. How does the xist activator Rlim/Rnf12 regulate xist expression? Biochem Soc Trans. 2024;52(3):1099–107. 38747697 10.1042/BST20230573 PMC11346418

[44] Luchsinger-Morcelle SJ, Gribnau J, Mira-Bontenbal H. Orchestrating Asymmetric Expression: Mechanisms behind Xist Regulation. Epigenomes, 2024. 8(1). 10.3390/epigenomes8010006 PMC10885031 38390897

[45] Penny GD, et al. Requirement for Xist in X chromosome inactivation. Nature. 1996;379(6561):131–7. 8538762 10.1038/379131a0

[46] Cui C, Shu W, Li P. Fluorescence in situ hybridization: cell-based genetic Diagnostic and Research Applications. Front Cell Dev Biol. 2016;4:89. 27656642 10.3389/fcell.2016.00089 PMC5011256

[47] Gozzetti A, Le Beau MM. Fluorescence in situ hybridization: uses and limitations. Semin Hematol. 2000;37(4):320–33. 11071355 10.1016/s0037-1963(00)90013-1

[48] Jonkers I, et al. Xist RNA is confined to the nuclear territory of the silenced X chromosome throughout the cell cycle. Mol Cell Biol. 2008;28(18):5583–94. 18625719 10.1128/MCB.02269-07 PMC2546918

[49] Lu Z, et al. RNA duplex map in living cells reveals higher-order transcriptome structure. Cell. 2016;165(5):1267–79. 27180905 10.1016/j.cell.2016.04.028 PMC5029792

[50] Lu Z, et al. Structural modularity of the XIST ribonucleoprotein complex. Nat Commun. 2020;11(1):6163. 33268787 10.1038/s41467-020-20040-3 PMC7710737

[51] Colognori D, et al. Xist Deletional Analysis reveals an interdependency between Xist RNA and polycomb complexes for spreading along the inactive X. Mol Cell. 2019;74(1):101–e11710. 30827740 10.1016/j.molcel.2019.01.015 PMC6469964

[52] Bousard A, et al. The role of Xist-mediated polycomb recruitment in the initiation of X-chromosome inactivation. EMBO Rep. 2019;20(10):e48019. 31456285 10.15252/embr.201948019 PMC6776897

[53] Pandya-Jones A, et al. A protein assembly mediates xist localization and gene silencing. Nature. 2020;587(7832):145–51. 32908311 10.1038/s41586-020-2703-0 PMC7644664

[54] Wutz A, Rasmussen TP, Jaenisch R. Chromosomal silencing and localization are mediated by different domains of Xist RNA. Nat Genet. 2002;30(2):167–74. 11780141 10.1038/ng820

[55] Chu C, et al. Systematic discovery of Xist RNA binding proteins. Cell. 2015;161(2):404–16. 25843628 10.1016/j.cell.2015.03.025 PMC4425988

[56] Chen CK, et al. Xist recruits the X chromosome to the nuclear lamina to enable chromosome-wide silencing. Science. 2016;354(6311):468–72. 27492478 10.1126/science.aae0047

[57] Engreitz JM, et al. The Xist lncRNA exploits three-dimensional genome architecture to spread across the X chromosome. Science. 2013;341(6147):1237973. 23828888 10.1126/science.1237973 PMC3778663

[58] Chaumeil J, et al. A novel role for xist RNA in the formation of a repressive nuclear compartment into which genes are recruited when silenced. Genes Dev. 2006;20(16):2223–37. 16912274 10.1101/gad.380906 PMC1553206

[59] Raposo AC, et al. The tandem repeat modules of Xist lncRNA: a Swiss army knife for the control of X-chromosome inactivation. Biochem Soc Trans. 2021;49(6):2549–60. 34882219 10.1042/BST20210253 PMC8786293

[60] Sarma K, et al. Locked nucleic acids (LNAs) reveal sequence requirements and kinetics of Xist RNA localization to the X chromosome. Proc Natl Acad Sci U S A. 2010;107(51):22196–201. 21135235 10.1073/pnas.1009785107 PMC3009817

[61] Nesterova TB, et al. Characterization of the genomic xist locus in rodents reveals conservation of overall gene structure and tandem repeats but rapid evolution of unique sequence. Genome Res. 2001;11(5):833–49. 11337478 10.1101/gr.174901 PMC311126

[62] Sakaguchi T, et al. Control of chromosomal localization of Xist by hnRNP. U Family Molecules Dev Cell. 2016;39(1):11–2. 27728779 10.1016/j.devcel.2016.09.022

[63] Sunwoo H, et al. Repeat E anchors xist RNA to the inactive X chromosomal compartment through CDKN1A-interacting protein (CIZ1). Proc Natl Acad Sci U S A. 2017;114(40):10654–9. 28923964 10.1073/pnas.1711206114 PMC5635913

[64] Ridings-Figueroa R, et al. The nuclear matrix protein CIZ1 facilitates localization of Xist RNA to the inactive X-chromosome territory. Genes Dev. 2017;31(9):876–88. 28546514 10.1101/gad.295907.117 PMC5458755

[65] Dixon-McDougall T, Brown CJ. Multiple distinct domains of human XIST are required to coordinate gene silencing and subsequent heterochromatin formation. Epigenetics Chromatin. 2022;15(1):6. 35120578 10.1186/s13072-022-00438-7 PMC8815261

[66] Nesterova TB, et al. Systematic allelic analysis defines the interplay of key pathways in X chromosome inactivation. Nat Commun. 2019;10(1):3129. 31311937 10.1038/s41467-019-11171-3 PMC6635394

[67] Gruenbaum Y, et al. The nuclear lamina comes of age. Nat Rev Mol Cell Biol. 2005;6(1):21–31. 15688064 10.1038/nrm1550

[68] Pombo A, Dillon N. Three-dimensional genome architecture: players and mechanisms. Nat Rev Mol Cell Biol. 2015;16(4):245–57. 25757416 10.1038/nrm3965

[69] Kind J, van Steensel B. Genome-nuclear lamina interactions and gene regulation. Curr Opin Cell Biol. 2010;22(3):320–5. 20444586 10.1016/j.ceb.2010.04.002

[70] Gjaltema RAF, et al. Distal and proximal cis-regulatory elements sense X chromosome dosage and developmental state at the xist locus. Mol Cell. 2022;82(1):190–e20817. 34932975 10.1016/j.molcel.2021.11.023

[71] Nesterova TB, et al. Skewing X chromosome choice by modulating sense transcription across the xist locus. Genes Dev. 2003;17(17):2177–90. 12952890 10.1101/gad.271203 PMC196458

[72] Dossin F, et al. SPEN integrates transcriptional and epigenetic control of X-inactivation. Nature. 2020;578(7795):455–60. 32025035 10.1038/s41586-020-1974-9 PMC7035112

[73] Robert-Finestra T, et al. SPEN is required for xist upregulation during initiation of X chromosome inactivation. Nat Commun. 2021;12(1):7000. 34853312 10.1038/s41467-021-27294-5 PMC8636516

[74] Lee JT, Lu N. Targeted mutagenesis of tsix leads to nonrandom X inactivation. Cell. 1999;99(1):47–57. 10520993 10.1016/s0092-8674(00)80061-6

[75] Sado T, Hoki Y, Sasaki H. Tsix silences xist through modification of chromatin structure. Dev Cell. 2005;9(1):159–65. 15992549 10.1016/j.devcel.2005.05.015

[76] Baran Y, et al. The landscape of genomic imprinting across diverse adult human tissues. Genome Res. 2015;25(7):927–36. 25953952 10.1101/gr.192278.115 PMC4484390

[77] Raudsepp T, Chowdhary BP. The Eutherian Pseudoautosomal Region. Cytogenet Genome Res. 2015;147(2–3):81–94. 26730606 10.1159/000443157

[78] Tukiainen T, et al. Landscape of X chromosome inactivation across human tissues. Nature. 2017;550(7675):244–8. 29022598 10.1038/nature24265 PMC5685192

[79] Cao R, et al. Role of histone H3 lysine 27 methylation in polycomb-group silencing. Science. 2002;298(5595):1039–43. 12351676 10.1126/science.1076997

[80] Zhao J, et al. Polycomb proteins targeted by a short repeat RNA to the mouse X chromosome. Science. 2008;322(5902):750–6. 18974356 10.1126/science.1163045 PMC2748911

[81] Almeida M, et al. PCGF3/5-PRC1 initiates polycomb recruitment in X chromosome inactivation. Science. 2017;356(6342):1081–4. 28596365 10.1126/science.aal2512 PMC6522364

[82] Zylicz JJ, et al. The implication of early chromatin changes in X chromosome inactivation. Cell. 2019;176(1–2):182–e19723. 30595450 10.1016/j.cell.2018.11.041 PMC6333919

[83] Sunwoo H, Wu JY, Lee JT. The xist RNA-PRC2 complex at 20-nm resolution reveals a low xist stoichiometry and suggests a hit-and-run mechanism in mouse cells. Proc Natl Acad Sci U S A. 2015;112(31):E4216–25. 26195790 10.1073/pnas.1503690112 PMC4534268

[84] Markaki Y, et al. Xist nucleates local protein gradients to propagate silencing across the X chromosome. Cell. 2021;184(25):6212. 34890555 10.1016/j.cell.2021.11.028 PMC8722464

[85] Monkhorst K, et al. X inactivation counting and choice is a stochastic process: evidence for involvement of an X-linked activator. Cell. 2008;132(3):410–21. 18267073 10.1016/j.cell.2007.12.036

[86] Lyon MF. Possible mechanisms of X chromosome inactivation. Nat New Biol. 1971;232(34):229–32. 5286191 10.1038/newbio232229a0

[87] de Barros ESL, et al. Kinetics of Xist-induced gene silencing can be predicted from combinations of epigenetic and genomic features. Genome Res. 2019;29(7):1087–99. 31175153 10.1101/gr.245027.118 PMC6633258

[88] Pasque V, Plath K. X chromosome reactivation in reprogramming and in development. Curr Opin Cell Biol. 2015;37:75–83. 26540406 10.1016/j.ceb.2015.10.006 PMC4688236

[89] Panning B, Jaenisch R. DNA hypomethylation can activate xist expression and silence X-linked genes. Genes Dev. 1996;10(16):1991–2002. 8769643 10.1101/gad.10.16.1991

[90] Qin S et al. Sex differences in the proliferation of pulmonary artery endothelial cells: implications for plexiform arteriopathy. J Cell Sci, 2020;133(9). 10.1242/jcs.237776 PMC7240306 32409569

[91] Carman BL et al. Dysregulation of the long noncoding RNA x-inactive-specific transcript expression in male patients with pulmonary arterial hypertension. Am J Pathol, 2024. 10.1016/j.ajpath.2024.04.005 PMC11284765 38705381

[92] Qin S, et al. Up-Regulation of the long noncoding RNA x-inactive-specific transcript and the sex Bias in Pulmonary arterial hypertension. Am J Pathol. 2021;191(6):1135–50. 33836164 10.1016/j.ajpath.2021.03.009 PMC8176134

[93] Sadagopan A, et al. Somatic XIST activation and features of X chromosome inactivation in male human cancers. Cell Syst. 2022;13(11):932–e9445. 36356577 10.1016/j.cels.2022.10.002

[94] Tower J. Sex-specific regulation of aging and apoptosis. Mech Ageing Dev. 2006;127(9):705–18. 16764907 10.1016/j.mad.2006.05.001

[95] Austad SN, Fischer KE. Sex differences in Lifespan. Cell Metab. 2016;23(6):1022–33. 27304504 10.1016/j.cmet.2016.05.019 PMC4932837

[96] Davis EJ, Lobach I, Dubal DB. Female XX sex chromosomes increase survival and extend lifespan in aging mice. Aging Cell. 2019;18(1):e12871. 30560587 10.1111/acel.12871 PMC6351820

[97] Hajdarovic KH, et al. Single-cell analysis of the aging female mouse hypothalamus. Nat Aging. 2022;2(7):662–78. 36285248 10.1038/s43587-022-00246-4 PMC9592060

[98] Chanda K, Mukhopadhyay D, Xist LRNA. X-chromosome instability and Alzheimer’s Disease. Curr Alzheimer Res. 2020;17(6):499–507. 32851944 10.2174/1567205017666200807185624

[99] Eldesouki S, et al. XIST in Brain Cancer. Clin Chim Acta. 2022;531:283–90. 35483442 10.1016/j.cca.2022.04.993

[100] Yang J, et al. Long non-coding RNA XIST: a novel oncogene in multiple cancers. Mol Med. 2021;27(1):159. 34930117 10.1186/s10020-021-00421-0 PMC8686246

[101] Dou DR, et al. Xist ribonucleoproteins promote female sex-biased autoimmunity. Cell. 2024;187(3):733–e74916. 38306984 10.1016/j.cell.2023.12.037 PMC10949934

[102] Miquel CH, Faz-Lopez B, Guery JC. Influence of X chromosome in sex-biased autoimmune diseases. J Autoimmun. 2023;137:102992. 36641351 10.1016/j.jaut.2023.102992

[103] Libert C, Dejager L, Pinheiro I. The X chromosome in immune functions: when a chromosome makes the difference. Nat Rev Immunol. 2010;10(8):594–604. 20651746 10.1038/nri2815

[104] Klein SL, Flanagan KL. Sex differences in immune responses. Nat Rev Immunol. 2016;16(10):626–38. 27546235 10.1038/nri.2016.90

[105] Wilkinson NM, et al. Sex differences in immunity. Annu Rev Immunol. 2022;40:75–94. 34985929 10.1146/annurev-immunol-101320-125133 PMC9805670

[106] Youness A, Miquel CH, Guery JC. Escape from X chromosome inactivation and the female predominance in Autoimmune diseases. Int J Mol Sci, 2021;22(3). 10.3390/ijms22031114 PMC7865432 33498655

[107] Sierra I, Anguera MC. Enjoy the silence: X-chromosome inactivation diversity in somatic cells. Curr Opin Genet Dev. 2019;55:26–31. 31108425 10.1016/j.gde.2019.04.012 PMC6759402

[108] Syrett CM, et al. Loss of Xist RNA from the inactive X during B cell development is restored in a dynamic YY1-dependent two-step process in activated B cells. PLoS Genet. 2017;13(10):e1007050. 28991910 10.1371/journal.pgen.1007050 PMC5648283

[109] Souyris M et al. TLR7 escapes X chromosome inactivation in immune cells. Sci Immunol, 2018;3(19). 10.1126/sciimmunol.aap8855 29374079

[110] Yu B, et al. B cell-specific XIST complex enforces X-inactivation and restrains atypical B cells. Cell. 2021;184(7):1790–e180317. 33735607 10.1016/j.cell.2021.02.015 PMC9196326

[111] Brown GJ, et al. TLR7 gain-of-function genetic variation causes human lupus. Nature. 2022;605(7909):349–56. 35477763 10.1038/s41586-022-04642-z PMC9095492

[112] Hornung V, et al. RNA recognition via TLR7 and TLR8. Handb Exp Pharmacol. 2008;183:71–86. 10.1007/978-3-540-72167-3_4 18071655

[113] Crawford JD et al. The XIST lncRNA is a sex-specific reservoir of TLR7 ligands in SLE. JCI Insight, 2023;8(20). 10.1172/jci.insight.169344 PMC10634230 37733447

[114] Nielsen MM, et al. Epigenetic and transcriptomic consequences of excess X-chromosome material in 47,XXX syndrome-A comparison with Turner syndrome and 46,XX females. Am J Med Genet C Semin Med Genet. 2020;184(2):279–93. 32489015 10.1002/ajmg.c.31799

[115] Scofield RH, et al. Klinefelter’s syndrome (47,XXY) in male systemic lupus erythematosus patients: support for the notion of a gene-dose effect from the X chromosome. Arthritis Rheum. 2008;58(8):2511–7. 18668569 10.1002/art.23701 PMC2824898

[116] De Vries GJ, et al. A model system for study of sex chromosome effects on sexually dimorphic neural and behavioral traits. J Neurosci. 2002;22(20):9005–14. 12388607 10.1523/JNEUROSCI.22-20-09005.2002 PMC6757680

[117] Syrett CM, et al. Loss of epigenetic modifications on the inactive X chromosome and sex-biased gene expression profiles in B cells from NZB/W F1 mice with lupus-like disease. J Autoimmun. 2020;107:102357. 31780316 10.1016/j.jaut.2019.102357 PMC7237307

[118] Hester J, Ventetuolo C, Lahm T. Sex, gender, and sex hormones in pulmonary hypertension and right ventricular failure. Compr Physiol. 2019;10(1):125–70. 31853950 10.1002/cphy.c190011 PMC7338988

[119] Predescu DN, Mokhlesi B, Predescu SA. The impact of sex chromosomes in the sexual dimorphism of pulmonary arterial hypertension. Am J Pathol. 2022;192(4):582–94. 35114193 10.1016/j.ajpath.2022.01.005 PMC8978209

[120] Tuder RM. Pathology of pulmonary arterial hypertension. Semin Respir Crit Care Med. 2009;30(4):376–85. 19634077 10.1055/s-0029-1233307

[121] Tuder RM. Pulmonary vascular remodeling in pulmonary hypertension. Cell Tissue Res. 2017;367(3):643–9. 28025704 10.1007/s00441-016-2539-y PMC5408737

[122] Pullamsetti SS, et al. Transcription factors, transcriptional coregulators, and epigenetic modulation in the control of pulmonary vascular cell phenotype: therapeutic implications for pulmonary hypertension (2015 Grover Conference series). Pulm Circ. 2016;6(4):448–64. 28090287 10.1086/688908 PMC5210074

[123] Crnkovic S, et al. Resident cell lineages are preserved in pulmonary vascular remodeling. J Pathol. 2018;244(4):485–98. 29359814 10.1002/path.5044 PMC5903372

[124] Navarro P, et al. Tsix-mediated epigenetic switch of a CTCF-flanked region of the xist promoter determines the xist transcription program. Genes Dev. 2006;20(20):2787–92. 17043308 10.1101/gad.389006 PMC1619945

[125] Chapman AG, et al. Differentially methylated CpG island within human XIST mediates alternative P2 transcription and YY1 binding. BMC Genet. 2014;15:89. 25200388 10.1186/s12863-014-0089-4 PMC4363909

[126] Migeon BR, et al. Identification of TSIX, encoding an RNA antisense to human XIST, reveals differences from its murine counterpart: implications for X inactivation. Am J Hum Genet. 2001;69(5):951–60. 11555794 10.1086/324022 PMC1274371

[127] Yang LG, et al. LncRNA XIST modulates HIF-1A/AXL signaling pathway by inhibiting mir-93-5p in colorectal cancer. Mol Genet Genomic Med. 2020;8(4):e1112. 32061057 10.1002/mgg3.1112 PMC7196477

[128] Bonnet S, et al. An abnormal mitochondrial-hypoxia inducible factor-1alpha-Kv channel pathway disrupts oxygen sensing and triggers pulmonary arterial hypertension in fawn hooded rats: similarities to human pulmonary arterial hypertension. Circulation. 2006;113(22):2630–41. 16735674 10.1161/CIRCULATIONAHA.105.609008

[129] Dai Z, et al. Prolyl-4 hydroxylase 2 (PHD2) Deficiency in endothelial cells and hematopoietic cells induces obliterative vascular remodeling and severe pulmonary arterial hypertension in mice and humans through hypoxia-inducible Factor-2alpha. Circulation. 2016;133(24):2447–58. 27143681 10.1161/CIRCULATIONAHA.116.021494 PMC4907810

[130] Lei W, et al. Expression and analyses of the HIF-1 pathway in the lungs of humans with pulmonary arterial hypertension. Mol Med Rep. 2016;14(5):4383–90. 27667582 10.3892/mmr.2016.5752

[131] Pullamsetti SS, et al. Hypoxia-inducible factor signaling in pulmonary hypertension. J Clin Invest. 2020;130(11):5638–51. 32881714 10.1172/JCI137558 PMC7598042

[132] Novoyatleva T, et al. Deficiency of Axl aggravates pulmonary arterial hypertension via BMPR2. Commun Biol. 2021;4(1):1002. 34429509 10.1038/s42003-021-02531-1 PMC8385080

[133] Cuthbertson I, Morrell NW, Caruso P. BMPR2 mutation and metabolic reprogramming in pulmonary arterial hypertension. Circ Res. 2023;132(1):109–26. 36603064 10.1161/CIRCRESAHA.122.321554

[134] Xie J, Long Noncoding RNAXIST. Regulates myocardial infarction via miR-486-5p/SIRT1 Axis. Appl Biochem Biotechnol. 2023;195(2):725–34. 36129595 10.1007/s12010-022-04165-3

[135] Xi L, et al. SIRT1 promotes pulmonary artery endothelial cell proliferation by targeting the akt signaling pathway. Exp Ther Med. 2020;20(6):179. 33101469 10.3892/etm.2020.9309 PMC7579766

[136] Zurlo G, et al. Sirtuin 1 regulates pulmonary artery smooth muscle cell proliferation: role in pulmonary arterial hypertension. J Hypertens. 2018;36(5):1164–77. 29369849 10.1097/HJH.0000000000001676

[137] Kostyunina DS, et al. Transcriptomics and proteomics revealed sex differences in human pulmonary microvascular endothelial cells. Physiol Genomics. 2024;56(2):194–220. 38047313 10.1152/physiolgenomics.00051.2023

[138] Bazan IS, et al. Sex differences and altered mitophagy in experimental pulmonary hypertension. Am J Physiol Lung Cell Mol Physiol. 2022;322(5):L761–9. 35137625 10.1152/ajplung.00019.2020 PMC9076415

[139] Tower J, Pomatto LCD, Davies KJA. Sex differences in the response to oxidative and proteolytic stress. Redox Biol. 2020;31:101488. 32201219 10.1016/j.redox.2020.101488 PMC7212483

[140] Bjork S, et al. Obstructive sleep apnea, obesity hypoventilation syndrome, and pulmonary hypertension: a state-of-the-art review. Sleep Med Clin. 2024;19(2):307–25. 38692755 10.1016/j.jsmc.2024.02.009

[141] Zhou Z, et al. LncRNA XIST promotes inflammation by downregulating GRalpha expression in the adenoids of children with OSAHS. Exp Ther Med. 2021;21(5):500. 33791009 10.3892/etm.2021.9931 PMC8005745

[142] Rouen A, et al. Multifactorial sleep disturbance in Klinefelter syndrome: a case report. Transl Androl Urol. 2023;12(7):1204–10. 37554521 10.21037/tau-22-587 PMC10406532

[143] Yamaguchi A, Knoblovits P. [Klinefelter syndrome and cardiovascular risk]. Hipertens Riesgo Vasc. 2018;35(4):195–8. 29398514 10.1016/j.hipert.2017.12.003

[144] Saito S, et al. [Klinefelter syndrome with congestive heart failure caused by sleep apnea]. Nihon Naika Gakkai Zasshi. 2003;92(6):1084–5. 12866457 10.2169/naika.92.1084

[145] Bianciardi E, et al. Laparoscopic sleeve gastrectomy for morbid obesity and Klinefelter syndrome: clinical report on two patients, with long-term follow-up. Eat Weight Disord. 2021;26(5):1685–90. 32654003 10.1007/s40519-020-00951-2

[146] Ramchandren S, Liebeskind DS. Headache in a patient with Klinefelter’s syndrome and hyperostosis frontalis interna. J Headache Pain. 2007;8(6):342–4. 18071629 10.1007/s10194-007-0426-3 PMC3476161

[147] Shanmugam VK, Tsagaris KC, Attinger CE. Leg ulcers associated with Klinefelter’s syndrome: a case report and review of the literature. Int Wound J. 2012;9(1):104–7. 21854549 10.1111/j.1742-481X.2011.00846.x PMC3664240

[148] Zore T, Palafox M, Reue K. Sex differences in obesity, lipid metabolism, and inflammation-A role for the sex chromosomes? Mol Metab. 2018;15:35–44. 29706320 10.1016/j.molmet.2018.04.003 PMC6066740

[149] Reue K. Sex differences in obesity: X chromosome dosage as a risk factor for increased food intake, adiposity and co-morbidities. Physiol Behav. 2017;176:174–82. 28284880 10.1016/j.physbeh.2017.02.040 PMC5444325

[150] Chen X, et al. The number of x chromosomes causes sex differences in adiposity in mice. PLoS Genet. 2012;8(5):e1002709. 22589744 10.1371/journal.pgen.1002709 PMC3349739

[151] Jiang-Feng M, et al. Prevalence and risk factors of diabetes in patients with Klinefelter syndrome: a longitudinal observational study. Fertil Steril. 2012;98(5):1331–5. 22940087 10.1016/j.fertnstert.2012.07.1122

[152] Aksglaede L, et al. Normal bone mineral content but unfavourable muscle/fat ratio in Klinefelter syndrome. Arch Dis Child. 2008;93(1):30–4. 17916585 10.1136/adc.2007.120675

[153] Ishikawa T, et al. Metabolic syndrome in men with Klinefelter’s syndrome. Urology. 2008;71(6):1109–13. 18455766 10.1016/j.urology.2008.01.051

[154] Bardsley MZ, et al. Insulin resistance and metabolic syndrome in prepubertal boys with Klinefelter syndrome. Acta Paediatr. 2011;100(6):866–70. 21251059 10.1111/j.1651-2227.2011.02161.x PMC4164507

[155] Gravholt CH, et al. Body composition, metabolic syndrome and type 2 diabetes in Klinefelter syndrome. Acta Paediatr. 2011;100(6):871–7. 21342256 10.1111/j.1651-2227.2011.02233.x

[156] Bojesen A, et al. The metabolic syndrome is frequent in Klinefelter’s syndrome and is associated with abdominal obesity and hypogonadism. Diabetes Care. 2006;29(7):1591–8. 16801584 10.2337/dc06-0145

[157] Hanna Kazazian N, et al. Lupus Autoimmunity and metabolic parameters are exacerbated upon high Fat Diet-Induced obesity due to TLR7 Signaling. Front Immunol. 2019;10:p2015. 10.3389/fimmu.2019.02015 PMC6738575 31552019

[158] Wu C, et al. Long noncoding RNA XIST regulates brown preadipocytes differentiation and combats high-fat diet induced obesity by targeting C/EBPalpha. Mol Med. 2022;28(1):6. 35062859 10.1186/s10020-022-00434-3 PMC8781062

[159] Shinozaki S, et al. Site-specific effect of estradiol on gene expression in the adipose tissue of ob/ob mice. Horm Metab Res. 2007;39(3):192–6. 17373633 10.1055/s-2007-970417

[160] Su Y, et al. Expression of long noncoding RNA xist is induced by glucocorticoids. Front Endocrinol (Lausanne). 2022;13:1005944. 36187119 10.3389/fendo.2022.1005944 PMC9516292

[161] Luijten IHN, et al. Glucocorticoid-Induced obesity develops independently of UCP1. Cell Rep. 2019;27(6):1686–98. e5. 31067456 10.1016/j.celrep.2019.04.041

[162] Parodi S. Xist noncoding RNA could act as a tumor suppressor gene in patients with classical Hodgkin’s disease. J Cancer Res Ther. 2020;16(1):7–12. 32362602 10.4103/jcrt.JCRT_1055_16

[163] Yildirim E, et al. Xist RNA is a potent suppressor of hematologic cancer in mice. Cell. 2013;152(4):727–42. 23415223 10.1016/j.cell.2013.01.034 PMC3875356

[164] Zhuang LK, et al. MicroRNA-92b promotes hepatocellular carcinoma progression by targeting Smad7 and is mediated by long non-coding RNA XIST. Cell Death Dis. 2016;7(4):e2203. 27100897 10.1038/cddis.2016.100 PMC4855645

[165] Farzaneh M, et al. Potential roles of lncRNA-XIST/miRNAs/mRNAs in human cancer cells. Clin Transl Oncol. 2023;25(7):2015–42. 36853400 10.1007/s12094-023-03110-y

[166] Fang J, Sun CC, Gong C. Long noncoding RNA XIST acts as an oncogene in non-small cell lung cancer by epigenetically repressing KLF2 expression. Biochem Biophys Res Commun. 2016;478(2):811–7. 27501756 10.1016/j.bbrc.2016.08.030

[167] Chen DL, et al. Long non-coding RNA XIST regulates gastric cancer progression by acting as a molecular sponge of miR-101 to modulate EZH2 expression. J Exp Clin Cancer Res. 2016;35(1):142. 27620004 10.1186/s13046-016-0420-1 PMC5020507

[168] Wassef M, Margueron R. The multiple facets of PRC2 alterations in cancers. J Mol Biol. 2017;429(13):1978–93. 27742591 10.1016/j.jmb.2016.10.012

[169] Yao Y, et al. Knockdown of long non-coding RNA XIST exerts tumor-suppressive functions in human glioblastoma stem cells by up-regulating miR-152. Cancer Lett. 2015;359(1):75–86. 25578780 10.1016/j.canlet.2014.12.051

[170] Agrelo R, et al. SATB1 defines the developmental context for gene silencing by Xist in lymphoma and embryonic cells. Dev Cell. 2009;16(4):507–16. 19386260 10.1016/j.devcel.2009.03.006 PMC3997300

[171] Chaligne R, et al. The inactive X chromosome is epigenetically unstable and transcriptionally labile in breast cancer. Genome Res. 2015;25(4):488–503. 25653311 10.1101/gr.185926.114 PMC4381521

[172] Dunford A, et al. Tumor-suppressor genes that escape from X-inactivation contribute to cancer sex bias. Nat Genet. 2017;49(1):10–6. 27869828 10.1038/ng.3726 PMC5206905

[173] Alkallas R, et al. Multi-omic analysis reveals significantly mutated genes and DDX3X as a sex-specific tumor suppressor in cutaneous melanoma. Nat Cancer. 2020;1(6):635–52. 35121978 10.1038/s43018-020-0077-8 PMC8832745

[174] Liu A, Liu L, Lu H. LncRNA XIST facilitates proliferation and epithelial-mesenchymal transition of colorectal cancer cells through targeting mir-486-5p and promoting neuropilin-2. J Cell Physiol. 2019;234(8):13747–61. 30656681 10.1002/jcp.28054 PMC13483007

[175] Li J, et al. Long noncoding RNA XIST: mechanisms for X chromosome inactivation, roles in sex-biased diseases, and therapeutic opportunities. Genes Dis. 2022;9(6):1478–92. 36157489 10.1016/j.gendis.2022.04.007 PMC9485286

[176] Marei HE, et al. p53 signaling in cancer progression and therapy. Cancer Cell Int. 2021;21(1):703. 34952583 10.1186/s12935-021-02396-8 PMC8709944

[177] Wise AL, Gyi L, Manolio TA. eXclusion: toward integrating the X chromosome in genome-wide association analyses. Am J Hum Genet. 2013;92(5):643–7. 23643377 10.1016/j.ajhg.2013.03.017 PMC3644627

